# Tuberculosis treatment adherence and associated factors in the Butha-Buthe district, Lesotho: a retrospective cohort study

**DOI:** 10.11604/pamj.2025.50.91.46218

**Published:** 2025-04-02

**Authors:** Motlatsi Rangoanana, Veranyuy Ngah, Jacques Lukenze Tamuzi, Sele Maphalale, Mabatho Molete, Retselisitsoe Ratikoane, Llang Maama, Isaac Fwemba, Olawande Daramola, Modupe Ogunrombi, Peter Suwirakwenda Nyasulu

**Affiliations:** 1 Division of Epidemiology and Biostatistics, Department of Global Health, Faculty of Medicine and Health Sciences, Stellenbosch University, Cape Town, South Africa,; 2 District Health Management Team of Butha-Buthe, Ministry of Health Lesotho, Maseru, Lesotho,; 3 National Tuberculosis Program, Ministry of Health Lesotho, Maseru, Lesotho,; 4 School of Public Health, University of Zambia, and Vanderbilt Medical Center University of Zambia, Lusaka, Zambia,; 5 Department of Informatics, Faculty of Engineering, Built Environment and IT, University of Pretoria, Pretoria, South Africa,; 6 Department of Clinical Pharmacology, Sefako Makgatho Health Sciences University, Pretoria, South Africa,; 7 Division of Epidemiology and Biostatistics, School of Public Health, Faculty of Health Sciences, Stellenbosch University, Cape Town, South Africa

**Keywords:** Tuberculosis burden, treatment adherence, drug resistance, rural population, Lesotho

## Abstract

**Introduction:**

Lesotho remains one of the world's 30 high-tuberculosis (TB) burden countries. In Butha-Buthe district, unfavourable TB treatment outcomes were higher than those set forth by the WHO. This study's objective was to evaluate TB treatment adherence and treatment resistance among patients enrolled in the 12 health facilities in Butha-Buthe.

**Methods:**

data were collected from the medical records of patients with sputum smear-positive TB and extra-pulmonary forms of TB between January 2015 and December 2020. Results were presented in frequencies and percentages. Univariate and multivariable logistic regression analyses were conducted to identify factors associated with treatment adherence.

**Results:**

among 1,792 patients who were enrolled, 1,320 were included in the study. The overall mean TB treatment adherence rate was estimated at 37.20%. Factors found to be associated with treatment adherence in multivariate analysis were age ≥60 years (aOR: 0.59, 95%CI: 0.54- 0.66; P<0.001), being a mine worker (aOR 1.09, 95%CI: 1.03-1.14; P<0.001), having pulmonary TB (aOR: 1.23, 95%CI: 1.17-1.29, P<0.001), being in the continuation phase of the treatment (aOR 1.38, 95%CI: 1.33, 1.45; P<0.001) and category 2 (aOR 0.93, 95%CI: 0.88-0.99; P = 0.016). Regarding TB contact support, family members (aOR: 1.08, 95%CI: 1.03-1.14; P<0.001), friends (aOR 1.30, 95%CI: 1.19-1.41; P<0.001), spouses (aOR: 1.24, 95%CI 1.16-1.34; P<0.001), and unreported contacts (aOR 1.18, 95%CI: 1.09-1.27; P = 0.015) all showed increased TB adherence.

**Conclusion:**

the overall adherence to TB therapy was poor in Butha-Buthe district. Lesotho urgently needs district-level strategies to improve TB treatment adherence and reduce treatment resistance.

## Introduction

Tuberculosis (TB) is the leading cause of death and the second highest cause of death from an infection after coronavirus disease 2019 (COVID-19) [1]. Tuberculosis caused more deaths than human immunodeficiency virus (HIV)/acquired immunodeficiency syndrome (AIDS), with a total of 1.3 million people dying from TB alone in 2022 [1]. Although the cumulative reduction in TB incidence was 11% in 2020, showing halfway to the End TB strategy milestone of 20% reduction, there was an increase in the global absolute numbers of TB deaths from 2019 to 2020, especially in most of the 30 high-TB-burden countries [2]. Relative to other World Health Organization (WHO) regions, sub-Saharan Africa has the highest burden of TB disease, with 29% of the 9 million TB cases worldwide [3].

Lesotho has the highest TB incidence globally, with an estimated 654 cases per 100,000 people annually [4]. The prevalence of multidrug-resistant TB (MDR-TB) is among the highest in sub-Saharan Africa, and HIV is a major driver of the TB epidemic, given the adult HIV prevalence of 21% [5]. The state of TB burden in Lesotho is worsened, given its burden of MDR-TB and extensively drug-resistant TB (XDR TB). This is further made complex by the country´s second-place rank for TB-HIV coinfection, estimated at 72% [6]. However, Lesotho is characterized by high unemployment, and widening inequalities have excluded most of the population from participation in economic development. The rural areas are home to most of the poor, and income distribution remains skewed in favour of the urban areas. This could have a direct impact on the healthcare sector and, more particularly, on TB management.

WHO recommends at least 85% to 90% treatment success rates for all TB diagnosed cases [7,8]. With early diagnosis and initiation of treatment that is completed, TB can be cured. However, factors such as late diagnosis and non-adherence to treatment are highly impactful on TB outcomes in Lesotho. In addition to this, the long duration of both drug-susceptible and drug-resistant TB treatment (at least 6 months) causes a challenge for patients to adhere to their treatment. Non-adherence to therapy has been cited as a major barrier to the control of TB [9]. A recent review reported that patient non-adherence is multidisciplinary and complex [10]. Similarly, non-adherence is a dynamic phenomenon influenced by a variety of interacting factors [11,12]. It is associated with higher transmission rates, morbidity, and costs of TB control programs [13]. Furthermore, it contributes to the persistence and resurgence of TB and is regarded as a major cause of relapse and drug resistance [12,14]. According to the literature, there is a scarcity of data on TB treatment adherence in Lesotho. Furthermore, a recent study conducted in Lesotho found significant staffing gaps in screening TB at health facilities across a district [15]. The objective of this study was to describe the burden of TB disease, treatment adherence, and drug resistance among the rural population in Lesotho.

## Methods

**Study design:** this was a retrospective record review of patients who were enrolled in TB treatment between January 2015 and December 2020.

**Population of study:** the study population consisted of male and female mine workers, ex-mine workers, and residents in the mining area who were diagnosed with TB. Patients with incomplete records, such as missing dates when treatment was started and finished and where such records could not be traced, were excluded from the study.

**Figure 1:**
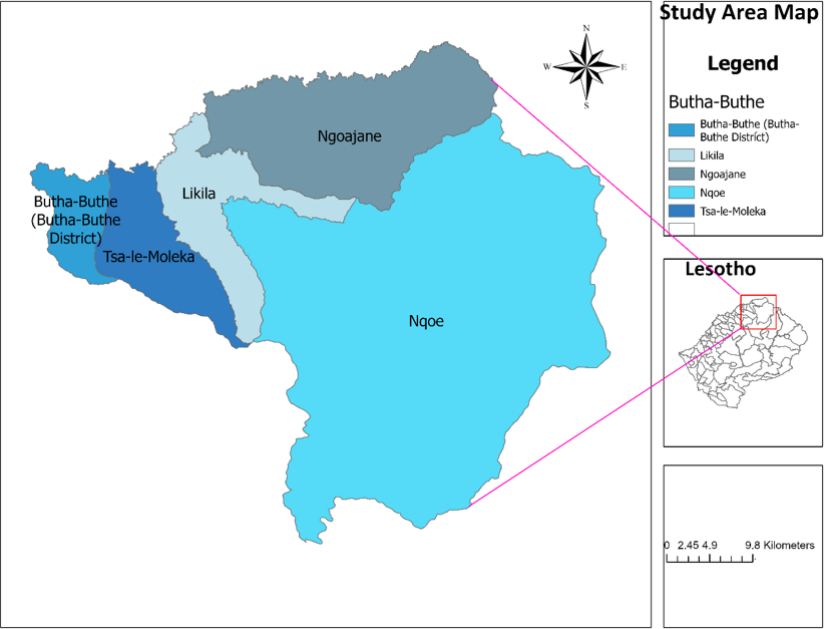
study area map

**Study setting:** the study was conducted in 12 health facilities (2 Hospitals and 10 Clinics) in Butha-Buthe district, Lesotho. These were the following facilities: Butha-Buthe Government Hospital and St Paul Clinic in Butha-Buthe district, Seboche Hospital and Tsime Clinic in Likila community, Ngoajane, Makhunoane, and Boiketsiso Clinics in Ngoajane community, Linakaneng Clinic in Tsa-le-Moleka community, Motete, Muela Clinic, St Peters Clinic, and Rampai Clinics in Nqoe community (Figure 1). The total estimated population for Butha-Buthe districts is around 105,785 (55,367 females and 50,418 males) [16]. Butha-Buthe reported the lowest HIV prevalence (79.1%) among the adults aged 15 and older during the population survey conducted in 2020 [17].

**Sample size estimation:** the national Lesotho TB adherence rate was reported to be 74.2% in 2016. Assuming the same adherence rate of 74.2% in a population of 105,785 at a 95% confidence interval and 80% power, we would require a sample size of 294.

**Data collection and management:** data on various exposure and outcome variables related to TB treatment were collected by data clerks from TB registers at the 12 health facilities (2 hospitals and 10 clinics) in Butha-Buthe district. Tuberculosis registers were those of TB patients´ records registered between 2015 and 2020. A team of data collectors reviewed the TB files and registers at the 12 facilities and entered data into an Excel spreadsheet. Data extracted included demographic information (age, sex, education, marital status, occupation), date of registration, type of TB disease, date treatment started and ended, phase of treatment, and treatment outcome (cured, lost to follow-up, death). Tuberculosis was categorized in four categories as described in Table 1. The data were cross-checked for errors and validated. Any ambiguous numbers were interrogated in consultation with the local people. Missing values were evaluated to see if they were missing at random or completely at random.

**Table 1:** tuberculosis classification by category

Cat 1	Tuberculosis exposure but no sign of infection. People in group 1 have been exposed to TB, yet their subsequent tuberculin skin tests are negative.
Cat 2	Latent TB infection but no disease. Cat 2 includes patients who have a positive tuberculin skin test but no symptoms or other signs of TB on a chest X-ray or other tests.
Cat 3	Persons who have active tuberculosis as determined by signs and symptoms and quick molecular diagnostic testing. Sputum smear microscopy, histopathology, and chest X-rays are among the other diagnostic methods available.
Cat 4	TB that is not clinically active. Tuberculosis is detected in patients who have previously had active tuberculosis but no longer have any evidence of the disease. Their skin tests are positive, and their chest X-rays may be abnormal, but they have no symptoms and negative lab results. Cat 4 TB refers to those who had active tuberculosis in the past but no longer have any evidence of the disease.

Cat: category; TB: tuberculosis

**Treatment adherence:** adherence was defined as ‘the extent to which the patient´s history of therapeutic drug taking coincides with the prescribed treatment [18]. There are two ways of measuring adherence in TB treatment, which are outcome-oriented (cure rate) and process-oriented (patient attendance to the facility, pill counts) [19]. These methods can be categorised into direct and indirect measures. The direct methods denote that treatment is taken under the supervision of an identified, trained, and supervised agent (health care provider, community volunteer, or any family members identified by the patient) who directly tracks the swallowing of anti-TB drugs [20]. Indirect methods entail patients self-reporting treatment outcome, which happens mostly at the facility level where questionnaires, interviews, scales, and patients´ diaries are being assessed [20]. We used treatment completion to measure TB treatment adherence. According to the Ministry of Health Lesotho, “TB treatment should be taken for at least 6 months (2 months of the intensive phase followed by 4 months of the continuation phase) for new TB cases [21].

**Data analysis:** analysis of the data was conducted using R and Integrated Nested Laplace Approximation (INLA) software packages. R was used for data cleaning, descriptive analysis, and fitting descriptive statistics. The INLA package was used for the Bayesian approaches to fit models with and without a random term (multilevel model). Demographic data was summarised using descriptive statistics. Person´s Chi-square test (X^2^) was used to investigate the associations between the patient´s characteristics and treatment adherence. Before multivariable analysis, bivariable analysis was undertaken, and independent variables with P< 0.20 were included in a multilevel multivariate regression. The adherence was modelled by multi-level logistic regression, with health facility as a random term that was specified to assume vague priors for the precision parameter. The model estimated independent variables´ fixed effects and included a random effect at the second level of analysis. It also included a random slope at the facility level. The estimates were obtained using the Bayesian modelling framework based on non-informative or weak prior information. To evaluate the adequacy of the multilevel model to the data, compared to the fixed-effect model, deviance information criteria were computed. The model that included random effect was found to be appropriately fitting the data. The odds ratios with their 95% confidence intervals were presented for both bivariable and multivariable analyses. A priori level of significance was set at p<0.05.

**Ethical approval:** this study was approved by Stellenbosch University's Human Research Ethics Committee and the Lesotho Ministry of Health (Ministry of Health Lesotho: 193-2019). Before reviewing patient files, written permission was obtained from the managers of the 12 health facilities.

## Results

**Figure 2:**
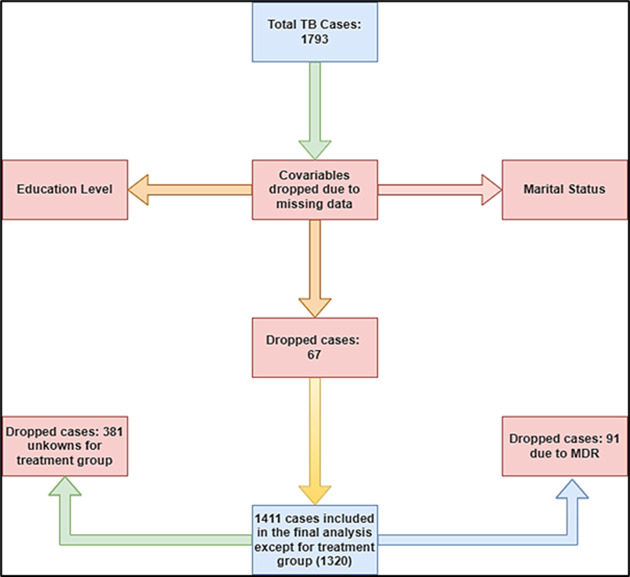
study flow diagram of tuberculosis treatment adherence in Butha-Buthe district/Lesotho

**Demographic characteristics:** among a total of 1,792 patients enrolled, 1,320 patients were included in the study (Figure 2). Table 2 presents the patients´ characteristics. About a third of the patients were female (n= 460, 343.8%), while 860 (65.2%) were their male counterparts. The 73.4% (n= 969 of the patients who were enrolled in the study were adults aged 20 and 59 years, while 4.17% (55) of patients were children below 20 years. Seboche hospital enrolled 902 of the patients (50.3%) followed by Butha-Buthe hospital with 410 patients (23%) with 7 patients (0.4%) reported from Motete. Most of the patients had a family member as their treatment contact (n=538, 40.8%). About seventy-one percent (n=939, 71.1%) of patients had pulmonary TB, while 28.9% (n=381) had extra-pulmonary TB. A great majority of the patients 774 (58.6%) were newly diagnosed in phase 1 of the treatment and were receiving treatment for the first time as category 1 treatment 1188 (90.0%).

**Table 2:** socio-demographic characteristics

Variables	Frequency (%)
	N=1320
**Sex**	
Female	460 (34.8%)
Male	860 (65.2%)
**Age category**	
<20 yrs	55 (4.17%)
20-59 yrs	969 (73.4%)
≥60 yrs	296 (22.4%)
**Occupation category**	
Employed	326 (24.7%)
Mine worker	356 (27.0%)
Unemployed	638 (48.3%)
**TB category**	
Extra-pulmonary tuberculosis	381 (28.9%)
Pulmonary tuberculosis	939 (71.1%)
**Treatment category**	
CAT 1	1188 (90.0%)
CAT 2	123 (9.32%)
CAT 3	9 (0.68%)
**Phase of treatment**	
Phase 1	774 (58.6%)
Phase 2	546 (41.4%)
**Treatment contact group**	
Community health worker	273 (20.7%)
Family member	538 (40.8%)
Friend	162 (12.3%)
Spouse	347 (26.3%)
**Adherent**	
Non-adherent	829 (62.8%)
Adherent	491 (37.2%)
**Death**	
Survived	1038 (78.6%)
Died	282 (21.4%)

CAT: category; TB: tuberculosis

**Figure 3:**
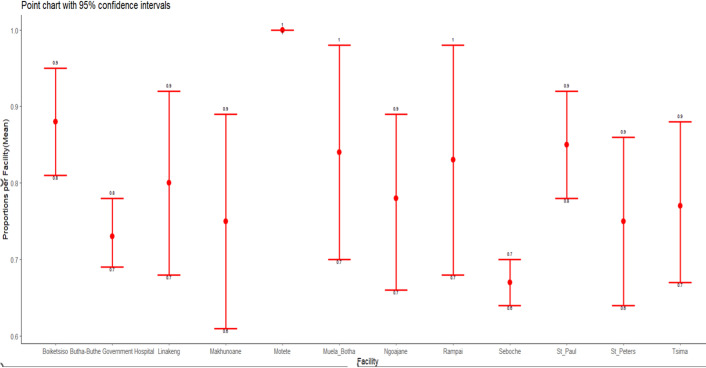
tuberculosis treatment adherence by facility

**Treatment adherence by demographic characteristics:** one thousand three hundred and twenty of the patients received treatment for at least 6 months (Table 3). Of this number, 74.7% (986) adhered to their TB treatment while 25.3% (334) patients did not adhere to their treatment (Table 3). Patients aged 20 - 59 have high TB adherence at 88.0% compared to other age groups. Patients who were less than 20 years old had the least TB adherence at 3.45% (Table 3). There was a statistically significant difference in adherence (< 0.001) between the different age groups. The differences in treatment adherence and non-adherence between the TB category, TB treatment phase, treatment contact group, and TB mortality were all statistically significant (P<0.001) (Table 3). Figure 3 reported TB treatment adherence by facility in Butha-Buthe district. Our results revealed relatively high TB treatment adherence (95%CI): Rampai clinic 70.83% (48.91%-87.38%), Seboshe hospital 42.46% (39.21%-45.76%), St Paul clinic 41.94% (31.78%-52.62%). However, Muela Botha, Linakeng, Motete, and Tsima clinics reported poor TB treatment adherence with 4.00% (0.10%-20.35%), 8.89% (2.48%-21.22%), 12.50% (0.32%-52.65%), and 14.52% (6.86%-25.78%) (Figure 3).

**Table 3:** comparison of socio-demographic characteristics of participants by treatment adherence

	Favorable outcome (adherent); N (%)	Unfavorable outcomes (non-adherent); N (%)	P-value
Total number	N=986	N=334	
**Sex**			0.989
Female	343 (34.80%)	117 (35.00%)	
Male	643 (65.20%)	217 (65.00%)	
**Age category**			<0.001
<20 yrs	34 (3.45%)	21 (6.29%)	
20-59 yrs	868 (88.00%)	101 (30.20%)	
≥60 yrs	84 (8.52%)	212 (63.50%)	
**Occupation category**			0.418
Employed	252 (25.6%)	74 (22.20%)	
Mine worker	260 (26.4%)	96 (28.70%)	
Unemployed	474 (48.1%)	164 (49.10%)	
**TB category**			<0.001
Extra-pulmonary TB	252 (25.60%)	129 (38.60%)	
Pulmonary TB	734 (74.40%)	205 (61.40%)	
**Treatment category**			0.257
CAT 1	881 (89.4%)	307 (91.9%)	
CAT 2	99 (10.0%)	24 (7.1%)	
CAT 3	6 (0.61%)	3 (1.00%)	
**Phase of treatment**			<0.001
Phase 1	463 (47.00%)	311 (93.10%)	
Phase 2	523 (53.00%)	23 (6.90%)	
**Treatment contact group**			<0.001
Community health Worker	176 (17.8%)	97 (29.0%)	
Family member	388 (39.4%)	150 (44.9%)	
Friend	137 (13.9%)	25 (7.50%)	
Spouse	285 (28.9%)	62 (18.6%)	
**Adherence**			<0.001
No	495 (50.2%)	334 (100%)	
Yes	491 (49.8%)	0 (0.00%)	
**Death**			<0.001
No	986 (100%)	52 (15.6%)	
Yes	0 (0.00%)	282 (84.4%)	

CAT: category; TB: tuberculosis

**Factors associated with TB treatment adherence:** in multivariate analysis, patients aged 60 years and above had a TB adherence reduction of 95% compared to those who were aged less than 20 years (aOR 0.05, 95% CI: 0.03-0.10) (Table 4). In contrast, TB treatment above 7 months was associated with increased adherence (aOR 2.18, 95%CI: 1.61-2.95). In contrast, miners were 1.09 times more likely (aOR1.09, 95% CI 1.03-1.14) to adhere to TB treatment compared to those who were unemployed. In the same line, pulmonary TB patients had a higher TB treatment adherence as compared to those with extra-pulmonary TB (EPTB) (aOR. 1.23, 95%CI: 1.17-1.29). Regarding TB contact, family members, friends, spouses, and unreported contacts all showed increased TB adherence with aOR (95%CI) 1.08(1.03, 1.14), 1.30(1.19-1.41), 1.24(1.16-1.34), and 1.18 (1.09, 1.27), respectively. Patients with TB in the continuation phase of their treatment were almost 1.38 times more likely to adhere to treatment (aOR=1.38, 95%CI: 1.33-1.45) compared to those who were in the intensive phase. Lastly, TB patients in Cat 2 were less likely to adhere to the treatment compared to Cat 1 (aOR 0.93, 95%CI: 0.88, 0.99) (Table 4).

**Table 4:** univariate and multivariate logistic regression analysis of factors associated with treatment adherence

Variable	Factors associated with TB treatment adherence			
	Unadjusted ORs (95% CI)	P-value	Adjusted ORs (95% CI)	P-value
**Age**				
<20 years	1		1	
20-59 years	1.23(1.12-1.35)	0.0013	1.00(0.92-1.10)	1.000
>60 years	0.71(0.64-0.78)	P<0.001	0.59(0.54-0.66)	P<0.001
**Occupation**				
Employed	1		1	
Mine worker	0.99 (0.93-1.04)	0.846	1.09(1.03-1.14)	P<0.001
Unemployed	0.98(0.93-1.03)	0.846	1.03(0.98-1.07)	1.000
**Sex**				
Female	1		1	
Male	1.04(0.99-1.08)	0.111	1.02(0.98-1.07)	0.0997
**TB category**				
Extra-pulmonary tuberculosis	1		1	
Pulmonary tuberculosis	1.08(1.02-1.14)	P<0.001	1.23(1.17-1.29)	P<0.001
**Treatment supporter**				
HCW	1		1	
Family member	1.07(1.00-1.14)	0.003	1.08(1.03-1.14)	P<0.001
Friend	1.15(1.04-1.28)	P<0.001	1.30(1.19-1.41)	P<0.001
Spouse	1.11(1.02-1.21)	P<0.001	1.24(1.16-1.34)	P<0.001
Unreported	1.05(0.95-1.15)	0.342	1.18(1.09-1.27)	0.0148
**Phase of Treatment**				
Phase 1	1		1	
Phase 2	1.51(1.44-1.58)	P<0.001	1.38(1.33-1.45)	P<0.001
**Treatment category**				
CAT 1	1		1	
CAT 2	1.05(0.98-1.13)	0.976	0.93(0.88-0.99)	0.016
CAT 3	1.08(0.88-1.33)	0.207	1.00(0.84-1.18)	1.000
CAT 4	1.14(0.84-1.56)	0.140	0.81(0.63-1.06)	0.115

CAT: category; HCW: health care worker; OR: odd ratio; TB: tuberculosis

**Description of tuberculosis resistance treatment:** in bivariate analysis of comparing favorable vs. unfavourable outcome for resistant TB treatment, age of <20 years, 20-59 years, and 60 years and above, TB phase 1, TB phase 2, non-adherent to treatment, adherent to treatment, survived, and died all reported a statistically significant P-value (Table 5). In bivariate analysis of comparing favorable vs. unfavourable outcome of being susceptible to TB treatment resistance, age of <20 years, 20-59 years, and 60 years and above, extra-pulmonary TB, pulmonary TB, TB phase 1, TB phase 2, non-adherent to treatment, adherent to treatment, survived, and died all reported a p-value of ≤0.05 (Table 6).

**Table 5:** bivariate analysis of treatment outcome for the cohort of patients that were reported to be resistant to tuberculosis treatment

	Favorable outcome	Unfavorable outcomes	P-value
	N=106	N=27	
**Sex**			0.783
Female	18 (17.0%)	5 (18.5%)	
Male	88 (83.0%)	22 (81.5%)	
**Age category**			<0.001
<20 yrs	5 (4.72%)	2 (7.4%)	
20-59 yrs	89 (84.0%)	10 (37.0%)	
≥60 yrs	12 (11.3%)	15 (55.6%)	
**Occupation category**			0.716
Employed	26 (24.5%)	7 (25.9%)	
Mine worker	44 (41.5%)	13 (48.1%)	
Unemployed	36 (34.0%)	7 (25.9%)	
**TB category**			0.102
Extra-pulmonary TB	31 (29.2%)	13 (48.1%)	
Pulmonary TB	75 (70.8%)	14 (51.9%)	
**Treatment category**			0.386
CAT 2	100 (94.3%)	24 (88.9%)	
CAT 3	6 (5.66%)	3 (11.1%)	
**Treatment phase**			<0.001
Phase 1	25 (23.6%)	24 (88.9%)	
Phase 2	81 (76.4%)	3 (11.1%)	
**Treatment contact group**			0.582
Community health workers	22 (20.8%)	6 (22.2%)	
Family member	37 (34.9%)	9 (33.3%)	
Friend	8 (7.55%)	0 (0.0%)	
Spouse	39 (36.8%)	12 (44.4%)	
**Adherence**			<0.001
Non adherent	55 (51.9%)	27 (100%)	
Adherent	51 (48.1%)	0 (0.00%)	
**Death**			<0.001
Survived	106 (100%)	3 (11.1%)	
Died	0 (0.00%)	24 (88.9%)	

CAT: category, TB: tuberculosis

**Table 6:** bivariate analysis of treatment outcome for the cohort of patients that were reported to be susceptible to TB treatment resistance

	Favorable outcome	Unfavorable outcomes	P-value
	N=881	N=308	
**Sex**			0.923
Female	325 (36.9%)	112 (36.4%)	
Male	556 (63.1%)	196 (63.6%)	
**Age category**			<0.001
<20 yrs	29 (3.29%)	19 (6.17%)	
20-59 yrs	780 (88.5%)	91 (29.5%)	
≥60 yrs	72 (8.17%)	198 (64.3%)	
**Occupation category**			0.395
Employed	227 (25.8%)	68 (22.1%)	
Mine worker	216 (24.5%)	83 (26.9%)	
Unemployed	438 (49.7%)	157 (51.0%)	
**TB category**			<0.001
Extra-pulmonary TB	221 (25.1%)	116 (37.7%)	
Pulmonary TB	660 (74.9%)	192 (62.3%)	
**Treatment category**			
CAT 1	881 (100%)	308 (100%)	
**Treatment phase**			<0.001
Phase 1	438 (49.7%)	287 (93.2%)	
Phase 2	443 (50.3%)	21 (6.8%)	
**Treatment contact group**			<0.001
**Community health workers**	155 (17.6%)	91 (29.5%)	
family member	351 (39.8%)	142 (46.1%)	
Friend	129 (14.6%)	25 (8.12%)	
Spouse	246 (27.9%)	50 (16.2%)	
**Adherence**			<0.001
Non-adherent	440 (49.9%)	308 (100%)	
Adherent	441 (50.1%)	0 (0.00%)	
**Death**			<0.001
Survived	881 (100%)	49 (15.9%)	
Died	0 (0.00%)	259 (84.1%)	

CAT: category; TB: tuberculosis

## Discussion

This study aimed to assess tuberculosis treatment adherence and resistance among patients at 12 health facilities in Butha-Buthe, Lesotho. Our results showed that TB adherence rates varied between health facilities in the Butha-Buthe district, with the lowest recorded in Seboche hospital (67%) and the highest in Motete clinic (100%). Our findings also revealed low treatment TB adherence in the Butha-Buthe district (37.20%), compared to the WHO target of 90%. The age 20-59 years, pulmonary TB, treatment contact (including family members, friends, spouses, and unreported), and treatment phase 2 were all associated with good TB treatment adherence. In contrast, the age of 60 was associated with poor TB treatment adherence. In contrast, mine workers, pulmonary TB, all treatment contacts, and phase 2 of treatment were associated with good TB adherence treatment. A low level of support and poor follow-up from the facility and community could be a plausible explanation. This could also be explained by the clinics' ineffective in-person Directly Observed Therapy (DOT) [21] and village tracking systems. All health facilities had lower TB adherence due to long distances, lack of transportation, missed appointments, lack of information provided, lack of community health workers, and tracking difficulties. Access to healthcare facilities has been identified as another factor that may influence TB adherence in other studies [22,23].

Tuberculosis adherence between age groups was statistically significant, with the 20-59-year-old age group having the highest adherence. This could be explained by the fact that this age group received more support for their treatment choices from family members, friends, community health workers, spouses, and other treatment supporters. In contrast, TB patients aged 60 years and up had lower adherence because they received less support. Our findings are consistent with previous research, which found that elderly patients had lower TB adherence due to physical infirmity, where they had to collect their medication every month, low income to level, comorbidities, and forgetfulness [20-24]. Besides, patients with pulmonary TB adhered better to treatment than patients with EPTB. Our result was in line with different studies showing unfavourable TB outcomes among EPTB patients [25]. Different studies reported suboptimal treatment success rates among EPTB patients [25-28]. Non-adherence to anti-TB drugs, potential side effects associated with anti-TB drugs, lack of patient knowledge about the consequences of loss to follow-up, and distance from treatment centre could all be reasons for the lower treatment success rate [25].

Our findings revealed that the choice of treatment support plays a significant role in TB treatment adherence. Tuberculosis patients who had treatment support from friends and spouses had higher adherence than other groups. In contrast, another study found that having at least one friend or family member was a borderline significant factor in TB treatment non-adherence [29]. According to a recent study, TB adherence is positively associated with the patient's education level, knowledge, family wealth, and provider-patient relationship [30]. Similarly, TB patients who chose their treatment supporters as friends had higher adherence than other groups. One possible explanation for this is the friends' openness and psychological support. Choosing community health care workers as treatment supporters, on the other hand, was associated with the lowest TB adherence to treatment. This could be explained by patients not feeling at ease with health care workers visiting their homes daily. Besides, TB Patients in the continuation phase had better treatment adherence compared to their counterparts in the initiation phase because of fewer side effects and improved health status in general. This was in line with a recent study showing that 65 (34.8%) of pulmonary TB and 45 (37.5%) of EPTB cases were adherent to TB treatment during the continuation phase [30].

In addition, studies have reported that the length of treatment plays a major role in TB adherence [20,21,24]. Compared to other employment sectors, mineworkers had a statistically significant TB adherence treatment history. Indeed, an estimated 500,000 mineworkers in South Africa's mines, approximately 40% of whom are from Southern African countries, including Lesotho [31]. The circular movement of mineworkers across provincial and national borders, as well as a poor cross-border health referral system, fuel infection rates, reduce adherence to TB treatment, and contribute to the sub-prevalence regions of drug-resistant strains such as MDR and XDR-TB [31]. According to studies, the prevalence of TB among miners is up to 10-15 times higher than in the general population [31-33]. Our findings suggest that mineworkers are better informed in this regard. In addition to this, multivariate logistic regression analysis also showed that TB category 2 had statistically poor adherence to the other categories. A study in India that looked at the clinical and demographic profile of defaulters as well as the reasons for stopping TB treatment among these retreatment patients in Category 2 found that side effects were the most common reason for treatment interruption [34].

Regarding drug-resistant TB, our results were consistent with studies that have demonstrated that the rate of drug-resistant TB was high in individuals with a history of prior anti-TB treatment, age, poor adherence to treatment, and a short duration of treatment [35-37]. Butha-Buthe district has a higher rate of drug-resistant TB (10.07%) than the global average of 3.2%. Efforts should be made to improve pre-treatment counseling, treatment by community-based DOTS providers, and short duration TB regimens, minimize serious side effects, and provide patients with repeated health education emphasizing the importance of continuing treatment in Butha-Buthe. This study could help public health policymakers implement healthcare facility-based interventions to improve TB treatment adherence in Butha-Buthe.

Our study had both strengths and weaknesses. Firstly, this is the first large-scale study of its kind in Lesotho, assessing TB treatment adherence. Second, the large sample size and multicenter characteristics, which included all of the settings in Butha-Buthe district, both rural and urban clinics. The study's main limitation was its retrospective record approach, which may have had an impact on data quality and completeness. Furthermore, missing data on HIV status, treatment adverse events, and comorbidities. Furthermore, the study did not include the adherence to X/MDR-TB.

## Conclusion

Butha-Buthe district has a greater rate of drug-resistant tuberculosis than the global average. Our study revealed that adherence to TB therapy was poor in the Butha-Buthe district. The age of 60 years and above, selection of treatment supporters, miners, treatment phase 2, treatment Cat 2, and pulmonary TB were all associated with TB adherence treatment. The study´s findings may be used as a baseline for modelling TB adherence and designing improved differentiated monitoring systems for patients enrolled in hospitals and clinics in Butha-Buthe district/Lesotho. Finally, effective strategies are required at the district level to mitigate TB treatment adherence factors and reduce drug resistance in Lesotho.

### What is known about this topic

- Lesotho's TB burden has worsened due to the prevalence of MDR-TB and extensively drug-resistant TB;
- Late diagnosis and non-adherence to treatment have a significant impact on tuberculosis outcomes in Lesotho;
- An unfavorable TB treatment outcome rate higher than that specified by the World Health Organization was recently observed in the Butha-Buthe district/Lesotho.

### What this study adds

- Butha-Buthe district/Lesotho had low TB treatment adherence;
- Age of 60 years or older, selection of treatment supporters, miners, TB treatment phase 2, TB treatment category 2, and pulmonary TB were all associated with TB adherence treatment;
- Butha-Buthe district has a higher rate of drug-resistant tuberculosis than the global averages.
